# Oral health barriers among community-dwelling older adults > =65 years with and without disability

**DOI:** 10.1093/geroni/igaf122.374

**Published:** 2025-12-31

**Authors:** Uma Kelekar, Debasree Das Gupta, Dinh Nguyen, Diep Tran

**Affiliations:** Marymount University, Arlington, Virginia, United States; Utah State University, Logan, Utah, United States; Unaffiliated, Frederick, Maryland, United States; Johns Hopkins University, Baltimore, Maryland, United States

## Abstract

Among US older adults ( > =65 years), multiple overlapping challenges, such as progressive aging, increasing comorbidities, and lack of universal dental coverage, restrict routine dental-care visits. The presence of one or more disabilities may further exacerbate access-related dental care disparities among the 65+. However, the literature on oral healthcare disparities within this population, particularly by disability status, remains limited. To address this gap, we employ 2012-2022 Medical Expenditure Panel Survey to 1) estimate dental service utilization rates among older adults with and without a disability, and 2) identify barriers associated with dental-care access among the 65+, after controlling for socio-demographics and health conditions. The main outcome, dental service utilization, was measured as a visit to a General Practitioner (GP) dentist in the last year. A secondary outcome represented barriers to dental care, measured by inability to access care/delaying care. Disability was measured as the presence of sensory, cognitive or physical limitations. In the multivariable analysis, in 2012, the odds of having seen a dentist (OR:0.68, 95% CI: 0.50,0.93) was lower among those with any disability compared to no disability. This difference between the 65+ with and without any disability was no longer significant in 2022. However, in 2022, the odds of reporting delays in seeking dental care [OR = 1.67, 95% CI: 1.13, 2.48] was higher among the 65+ reporting any disability, compared to those reporting no disability. Oral health disparities among older adults with disabilities continue to persist and should be addressed through adequate policy reform and investments in health education programs.

